# The Mastic Tree (*Pistacia lentiscus* L.) Leaves as Source of BACs: Effect of Growing Location, Phenological Stage and Extraction Solvent on Phenolic Content

**DOI:** 10.17113/ftb.58.03.20.6662

**Published:** 2020-09

**Authors:** Sanja Dragović, Verica Dragović-Uzelac, Sandra Pedisić, Zrinka Čošić, Maja Friščić, Ivona Elez Garofulić, Zoran Zorić

**Affiliations:** 1 IREKS AROMA Ltd., Trešnjevka 24, HR-10450 Jastrebarsko, Croatia; 2 Faculty of Food Technology and Biotechnology, University of Zagreb, Pierottijeva 6, HR-10000 Zagreb, Croatia; 3 Faculty of Pharmacy and Biochemistry, University of Zagreb, Schrottova 39, HR-10000 Zagreb, Croatia

**Keywords:** mastic tree leaves, growing location, phenological stage, phenolic concentration, extraction solvent

## Abstract

**Research background:**

Mastic tree (*Pistacia lentiscus* L.) of the Anacardiaceae family is an evergreen shrub from Mediterranean countries where it is used in traditional medicine. Analysis of *P. lentiscus* leaf, stem, fruit and root extracts showed high concentrations of principal groups of secondary metabolites (flavonoids, phenolic acids and tannins), suggesting the plant possesses great biological potential. Therefore, the aim of this research is to evaluate the impact of environmental parameters and the extraction solvent type on the concentration of phenols in mastic tree leaf extracts grown at four different locations along the Adriatic coast (Barbariga, Lun, Hvar and Vela Luka) during three phenological stages (early flowering, early fruiting and late fruiting).

**Experimental approach:**

Since mastic tree plant has phenolic compounds with different structures and chemical properties, ethanolic and methanolic leaf extracts were analysed using high-performance liquid chromatography (HPLC) coupled with UV/Vis PDA detector. Phenolic compounds were identified by comparing the retention times and spectral data with those of standards at 280 and 340 nm.

**Results and conclusions:**

In all samples, phenolic acids and flavonol glycosides were quantified, while catechin was quantified only in methanolic extracts. The 5-*O*-galloylquinic acid was determined as a predominant phenolic compound in all samples followed by monogalloyl glucose, 3,5-di-*O*-galloylquinic acid, 3,4,5-tri-*O*-galloylquinic acid and gallic acid, respectively. Myricetin-3-*O*-rhamnoside was found to be the predominant flavonol glycoside followed by myricetin-3-*O*-glucoside, myricetin-3-*O*-glucuronide, quercetin-3-*O*-rhamnoside and derivative of flavonol glycoside. The mass concentration of these compounds significantly varied during different phenological stages, at different growing locations and used extraction solvents. The highest phenolic mass concentration was determined in the samples harvested at Hvar growing location and extracted in 80% methanol. The highest total phenolic acid mass concentration was obtained in the samples harvested during the flowering phenological stage and the highest total flavonoid mass concentration in the samples harvested during the early fruiting stage.

**Novelty and scientific contribution:**

The obtained data provide a better understanding of the *P. lentiscus* species phenolic concentration, which can lead to further investigations regarding the valorisation of mastic tree leaves as pharmaceutical products or as food products with added value.

## INTRODUCTION

Mastic tree (*Pistacia lentiscus* L.) is an evergreen shrub from Anacardiaceae family (*1*) widely spread throughout the Mediterranean countries, including Republic of Croatia, where it grows along the Adriatic coast and on the islands. The mastic tree plant (roots, leaves, branches and berries) is often applied in traditional medicine for treatment of gastrointestinal diseases, eczema and throat infections (*2*-*4*) due to strong antioxidant (*5*), anti-inflammatory and antimicrobial effects (*6*, *7*). These health-promoting properties of mastic tree have been attributed to the presence of various biologically active compounds (BACs) such as phenolic compounds (*8*). Phenolic compounds are secondary metabolites that contain one or more aromatic rings joined with one or more hydroxyl groups in their basic structure and are distributed in different classes, such as flavonoids, tannins, stilbenes, phenolic acids and lignans (*9*).

The phytochemical analysis of leaf, stem, fruit and root extracts of *P. lentiscus* showed the presence of principal groups of secondary metabolites (flavonoids, phenolic acids and tannins) (*10*). A study on Algerian mastic tree showed that total phenolic mass fraction in leaves as gallic acid equivalents (GAE) on dry matter basis ((216.28±20.62) mg/g was significantly higher than that in stems ((121.39±3.35) mg/g), fruits ((103.34±2.32) mg/g) and roots ((30.18±1.29) mg/g) (*10*). Mastic tree leaves had the highest phenolic content, with the most abundant compounds being flavonoids, myricetin glycoside, catechin, β-glucogallin, quercitrin-*O*-gallate and gallic and 5-*O*-galloylquinic acids (*7*).

Phenolics are produced by various biosynthetic pathways including the action of intact enzymatic complexes, but their quantity and composition change during different phenological stages. Differences in the phenolic content obtained from various parts of the plant mainly depend on genetic origin, but stressful environmental conditions such as temperature (difference between night and day), soil fertility, moisture content, light, geographical origin and phenological stage (*11*-*14*) can significantly redirect metabolism towards the production of secondary plant metabolites with higher bioactivity. There are numerous studies on different plant material concerning the optimal phenological stage and the influence of environmental factors on phenolic compound yields (*15*, *16*). The link between the accumulation of secondary plant metabolites due to environmental stresses and different stimulants has not been fully defined yet. To obtain maximum yields of targeted BACs and their stability, it is also very important to select the optimal extraction method. Numerous studies have proven the advantages of novel extraction techniques (microwave-assisted extraction (MAE), supercritical extraction (SCE) and ultrasound-assisted extraction (UAE)) over conventional ones (*e.g*. maceration or Soxhlet) (*17*, *18*) due to their low-cost and availability. However, conventional extraction methods are more successful for phenolic compound isolation than novel techniques, despite the high solvent requirements, time and energy consumption (*19*). Vujanović *et al*. (*18*) compared the efficacy of the extraction of phenolic compounds from *Sambucus nigra* L. flowers using maceration, UAE and MAE, and stated that macerated extract contained phenolic compounds that were absent from the extracts obtained by UAE and MAE. For each extraction method, conventional or novel, optimization of extraction parameters such as the choice of solvent, extraction time and temperature is necessary. Various studies indicate that the polarity of the solvent plays an important role in the BAC yield from plant matrices, while solvents are usually based on aqueous mixtures containing ethanol, methanol, acetone or ethyl acetate (*20*). For example, different parts of *P. lentiscus* (leaves, stems, fruits and roots) were extracted with different solvents and higher yields of BACs were found in methanolic fractions than in ethyl acetate and butanolic fractions (*10*). Haas *et al*. (*21*) also reported that the highest phenolic concentration from grape residue was obtained by 80% methanol/water solution compared to 80% ethanol or acetone-water solution.

Therefore, we can summarize that great variability in phenolic composition and quantity could be attributed to the plant origin and phenological stage, environmental conditions, methods of extraction and to the polarity of used organic solvents.

Although mastic tree leaves have a great biological potential, the literature data considering the influence of phenological stage and environmental factors on the accumulation of secondary plant metabolites in mastic tree leaves is scarce, as well as the extraction efficiency and yields of phenolic compounds (*22*). Mastic tree leaf certainly has the potential for developing nutraceuticals and dietary supplements and thus it is essential to determine the composition and quantities of its derived phenolic compounds during phenological stages.

Therefore, the objectives of this study are as follows: (*i*) to compare the effects of different growing locations and phenological stages on the accumulation, composition and content of phenolic compounds in mastic tree leaves during vegetation, and (*ii*) to examine the influence of two solvent types on the extraction efficiency and yields of phenolic compounds.

## MATERIALS AND METHODS

### Plant material

Aerial parts (leaves and branches) of *Pistacia lentiscus* tree were harvested in 2019 along the Adriatic coast at four different locations (Barbariga, Lun, Hvar and Vela Luka), during three phenological stages: early flowering in May, early fruiting in August, and late fruiting stage in October, always taken from the same tree (Table 1). Plant material was identified by using usual keys and iconographies with support of the Department of Pharmaceutical Botany, Faculty of Pharmacy and Biochemistry, University of Zagreb, Croatia. The samples were cleaned to remove damaged branches and leaves, air-dried in dry air oven (FN 500; Nüve, Ankara, Turkey) to constant mass and ground to a fine powder with a mill (Nutribullet, Capital Brands LLC, Los Angeles, CA, USA). Approximately 20 g of *P. lentiscus* leaf powder were packed in bags (15 cm×15 cm) of commercially available laminates - PET/PPmet/PE (Folijaplast Ltd, Zadar, Croatia) and afterwards stored at -18 °C until further analysis.

**Table 1:** Geographic coordinates, phenological stages and bioclimatic characteristics of the growing locations of *Pistacia lentiscus* L. samples in 2019

Growing location	Altitude/m	Latitude	Longitude	Phenological stage	Average temperature/°C	Average insolation/h	Average precipitation/mm
Barbariga		44°59'27''N	13° 44' 12''E	1	17.2	311.7	84.2
	4			2	24.6	352.9	51.5
				3	14.4	174.4	27.7
Lun		44°40'59''N	14°45'15''E	1	18.9	306.7	48.7
	15			2	26.9	358.2	7.00
				3	16.1	199.9	40.5
Hvar		43°7'51''N	16°56'34''E	1	19.7	318.1	33.3
	330			2	27.8	368.4	0.00
				3	17.5	243.1	25.3
Vela Luka		42°57'40''N	16°43'17''E	1	18.7	289.4	19.3
	45			2	26.4	384.4	0.00
				3	15.2	230.2	27.8

1=early flowering stage (May, 2019), 2=early fruiting stage (August, 2019), 3=late fruiting stage (October, 2019)

### Climatic data

Climatic data (Table 1) from the growing locations were provided by the Croatian Meteorological and Hydrological Service (CMHS). Interpretation of the results was based on the daily average maximum and minimum temperatures and mean precipitation through the month in which the samples were harvested.

### Chemicals

Solvents (methanol and ethanol) and reagents used in the extractions were of analytical grade and purchased from Kemika (Zagreb, Croatia). Solvents used for mobile phases (formic acid and acetonitrile) were of HPLC grade, purchased from BDH Prolabo, VWR (Lutterworth, UK). Water was Milli-Q quality (Millipore Corp., Bedford, MA, USA). Standards, quercetin-3-*O*-glucoside, gallic, caffeic and chlorogenic acid were purchased from Sigma-Aldrich, Merck (Steinheim, Germany).

### Conventional extraction of phenolic fractions

Phenolic compounds were extracted from the prepared plant material ((2.000±0.001) g) with 20 mL of 80% aqueous solution of methanol or ethanol, which was facilitated by shaking at room temperature and 112×*g* (VXR Vibrax; IKA, Königswinter, Germany). The extracts were filtered through Whatman No. 40 filter paper (Whatman International Ltd., Kent UK). Thereafter, the extracts were stored at -15 °C for further analysis (not longer than 7 days). The obtained phenolic compound extracts were used for determination of total phenolic acids (TPA), total flavonol glycosides (TFG) and individual phenolic compounds using HPLC coupled with UV/Vis PDA detector (Agilent Technologies, Santa Clara, CA, USA).

### HPLC analysis of phenolic compounds in the extracts

Phenolic compounds were analysed by a direct injection of the extracts, previously filtered through a 0.45-µm pore size membrane filter (Macherey-Nagel GmbH & Co. KG, Düren, Germany). Chromatographic separation was performed using HPLC analysis with Agilent 1260 quaternary LC Infinity system (Agilent Technologies) equipped with diode array detector (DAD), an automatic injector and ChemStation software.

The compounds were separated on a Luna 100-5C18, 5 µm (250 mm×4.6 mm i.d.) column (Phenomenex, Aschaffenburg, Germany). The solvent composition and the used gradient conditions were described previously by Fecka and Turek (*23*) with some modifications: instead of 0.2, 1.5 and 5% solvent A (acetonitrile) and B (water), the solvent contained 3% formic acid. The used elution program was as follows: 90% B at 0 min, and then 60% B from 0 to 25 min, 70% B from 25 to 30 min, and 10% B from 30 to 35 min. The flow rate was 0.9 mL/min and the injection volume 20 µL.

Detection was performed with UV/VIS–photodiode array detector by scanning from 220 to 380 nm. Phenolic compounds were identified by comparing the retention times and spectral data with those of the authentic standards at 280 and 340 nm. Quantifications were made by the external standard method.

Quantitative determination was carried out using the calibration curves of the standards (gallic acid: y=30.025x, R^2^=0.99; quercetin-3-*O*-glucoside: y=37.386x, R^2^=1.00. Monogalloyl glucose (MG-Glu), 5-*O*-galloylquinic acid (5-GQA) and 3,5-di-*O*-galloylquinic acid (3,5-diGQA) were determined as gallic acid equivalents, and myricetin-3-*O*-glucuronide (My-G), myricetin-3-*O*-glucoside (My-Glu), myricetin-3-*O*-rhamnoside (My-R) and quercetin-3-*O*-rhamnoside (Que-R) as quercetin-3-*O*-glucoside equivalents.

### Statistical analysis

Data analyses were performed using the Statistica v. 10.0 (*24*). All measurements were performed in triplicate and the results are presented as mean value±standard deviation (S.D.). In order to explore the influence of growing location, phenological stage and extraction solvent, analysis of variance (factorial ANOVA) was carried out and marginal mean values were compared with Tukey’s honestly significant difference (HSD) test.

## RESULTS AND DISCUSSION

Plants constantly face biotic and abiotic stresses during their life cycle (plant growth, flower development, seed maturing) resulting in chemical composition changes and biosynthesis of secondary plant metabolites such as phenolic compounds (*25*). Seasonal influence attributed to climatic conditions such as temperature and precipitation have a considerable impact on the accumulation of plant phenolics since they play a crucial role in plant adaptation and protection (*26*). Phenolic compounds are present in many plant species including *Pistacia lentiscus* leaves, which are an abundant source of flavonoids, phenolic acids and their derivatives (*7*). An efficient extraction procedure, *i.e.* the proper solvent selection, also has a significant impact on the precise separation and quantification of the phenolic compounds.

In this study, mastic tree leaves were harvested along the Adriatic coast at four different growing locations (Barbariga, Lun, Hvar and Vela Luka) during three phenological stages. Phenolic compounds were extracted with 80% methanol and 80% ethanol to determine the influence of these solvents on the extraction yield of phenolic compounds.

The composition and concentrations of phenolic compounds in the methanolic and ethanolic extracts of mastic tree leaves are shown in Table 2 and Table 3. In the analysed *P. lentiscus* leaf extracts, seven phenolic acids (monogalloyl glucose, gallic acid, 5-*O*-galloylquinic acid, 3,5-di-*O*-galloylquinic acid, 3,4,5-tri-*O*-galloylquinic acid, caffeic acid and caffeic acid derivate), five flavonol glycosides (myricetin-3-*O*-glucuronide, myricetin-3-*O*-glucoside, myricetin-3-*O*-rhamnoside, quercetin-3-*O*-rhamnoside and flavanol-glycoside derivative 1) and flavanol catechin were determined, which is in accordance with previous studies (*22*). Results found in our research are in a close agreement with those reported by Rodriguez-Perez *et al*. (*27*).

**Table 2:** The mass concentration of phenolic acids in the extracts of mastic tree leaves harvested at four different locations along the Croatian Adriatic coast during three phenological stages

	Growing location	Phenological stage	MG-Glu	GA	5-GQA	3,5-diGQA	3,4,5-tGQA	CA	CA (der.)	TPA
	*γ*/(mg/L)							
*φ*(methanol)=80%	Barbariga	1	(54.3±0.3)^j^	(27.5±0.1)^c,d,e^	(106.4±0.4)^o^	(22.4±0.3)^d,e,f,g^	(57.5±0.4)^p^	(10.3±0.2)^e^	(0.91±0.03)^b^	(279.3±0.4)^q^
		2	(32.5±0.5)^d^	(16.5±0.3)^a,b,c^	(114.5±0.2)^p^	(21.3±0.4)^d,e,f,g^	(20.2±0.4)^e,f^	tr	tr	(205.2±1.6)^m^
		3	(28.9±0.3)^b,c^	(8.1±0.3)^a,b,c^	(48.5±0.4)^e^	(31.6±0.2)^g,h^	(19.6±0.2)^e^	tr	tr	(136.8±0.5)^e^
	Lun	1	(69.3±0.6)^l^	tr	(53.7±0.3)^f^	(32.4±0.5)^g,h^	(25.4±0.1)^j^	(2.5±0.2)^c^	tr	(183.4±0.8)^l^
		2	(30.2±0.6)^c^	(30.3±0.5)^c,d,e^	(130.6±0.3)^q^	(18.4±0.3)^c,d,e,f^	(10.6±0.3)^b^	tr	tr	(220.1±1.2)^n^
		3	(33.1±0.2)^d^	(33.6±0.3)^d,e^	(129.3±0.2)^q^	(24.5±0.3)^e,f,g^	(21.3±0.4)^f,g^	tr	tr	(241.9±0.3)^o^
	Hvar	1	(96.3±0.3)^p^	(25.5±0.3)^c,d,e^	(186.5±0.4)^t^	(95.5±0.2)^k^	(53.5±0.2)^o^	tr	tr	(457.2±1.1)^z^
		2	(92.4±0.3)^o^	(30.1±0.3)^c,d,e^	(137.4±0.3)^r^	(69.6±0.4)^x^	(23.3±0.4)^h,i^	tr	tr	(352.8±0.2)^t^
		3	(50.4±0.4)^i^	(26.7±0.4)^c,d,e^	(99.5±0.4)^m^	(52.0±0.2)^j^	(38.5±0.4)^m^	tr	tr	(267.2±0.9)^p^
	Vela Luka	1	(108.5±0.1)^q^	(24.5±0.3)^c,d,e^	(147.3±0.5)^s^	(92.3±0.3)^k^	(43.5±0.4)^n^	(0.78±0.03)^a^	tr	(416.9±0.5)^v^
		2	(42.4±0.3)^g^	(16.4±0.5)^a,b,c^	(67.4±0.4)^i^	(28.4±0.3)^f,g^	(22.5±0.4)^g,h^	tr	tr	(177.1±0.8)^k^
		3	(38.5±0.4)^e,f^	(21.9±0.1)^a,b,c,d,e^	(83.5±0.2)^k^	(16.4±0.4)^b,c,d,e^	(12.6±0.2)^c^	tr	tr	(172.9±0.7)^j^
*φ*(ethanol)=80%	Barbariga	1	(37.3±0.2)^e^	(26.5±0.3)^c,d,e^	(59.4±0.2)^h^	tr	(24.4±0.2)^i,j^	tr	tr	(147.6±0.4)^f^
		2	(27.8±0.2)^b^	tr	(19.5±0.4)^b^	(12.6±0.4)^b,c,d^	(13.2±0.2)^c^	tr	tr	(73.2±0.6)^b^
		3	(5.4±0.3)^a^	(0.45±0.04)^d^	(2.6±0.4)^a^	tr	(0.67±0.03)^a^	(7.6±0.4)^d^	(0.58±0.03)^a^	(17.31±0.52)^a^
	Lun	1	(43.4±0.4)^g^	(6.5±0.4)^a,b^	(22.5±0.4)^c^	(13.5±0.4)^b,c,d,e^	(19.5±0.3)^e^	(1.58±0.03)^b^	tr	(106.9±0.5)^c^
		2	(28.4±0.2)^b^	(19.3±0.4)^a,b,c,d,e^	(53.4±0.3)^f^	tr	(26.7±0.3)^k^	tr	tr	(127.7±0.2)^d^
		3	(43.5±0.3)^g^	(26.3±0.3)^c,d,e^	(66.6±0.4)^i^	(9.5±0.4)^a,b,c^	(23.33±0.09)^h,i^	tr	tr	(169.3±1.0)^i^
	Hvar	1	(79.6±0.4)^n^	(66.6±0.2)^f^	(104.4±0.7)^n^	(47.2±0.1)^i,j^	(73.6±0.5)^s^	tr	tr	(371.4±1.5)^u^
		2	(71.5±0.3)^m^	(51.3±0.2)^f^	(91.5±0.3)^l^	(40.6±0.3)^h,i^	(69.2±0.2)^r^	tr	tr	(324.1±1.1)^s^
		3	(39.3±0.3)^f^	(21.5±0.3)^a,b,c,d,e^	(55.6±0.3)^g^	(19.1±0.3)^c,d,e,f^	(44.5±0.4)^n^	(0.62±0.05)^a^	tr	(180.6±0.9)^l^
	Vela Luka	1	(58.1±0.2)^k^	(55.5±0.4)^f^	(80.5±0.3)^j^	(48.0±0.2)^i,j^	(67.5±0.4)^q^	tr	tr	(309.6±2.3)^r^
		2	(49.6±0.2)^i^	(16.6±0.3)^a,b,c,d^	(14.6±0.3)^b,c,d,e^	(14.6±0.3)^b,c,d,e^	(34.6±0.3)^l^	tr	tr	(157.6±0.6)^h^
		3	(45.3±0.2)^h^	(24.4±0.3)^b,c,d,e^	(58.4±0.3)^h^	(5.5±0.3)^a,b^	(17.7±1.2)^d^	tr	tr	(151.2±2.2)^g^

The results in each row marked with the same letter do not differ statistically at p≤0.05.

MG-Glu=monogalloyl glucose, GA=gallic acid, 5-GQA=5-*O*-galloyl-quinic acid, 3,5-diGQA=3,5-di-*O*-galloyl-quinic acid, 3,4,5-tGQA=3,4,5-tri-*O*-galloyl-quinic acid, CA=caffeic acid, CA (der.)=caffeic acid derivative, TPA=total phenolic acid, tr=traces

1=early flowering stage (May, 2019), 2=early fruiting stage (August, 2019), 3=late fruiting stage (October, 2019)

**Table 3:** The mass concentration of flavonol glycosides and catechin in the extracts of mastic tree leaves harvested at four different locations along the Croatian Adriatic coast during three phenological stages

	Growing location	Phenological stage	My-G	My-Glu	My-R	Que-R	F-G der 1	TFG	Catechin
*γ*/(mg/L)						
*φ*(methanol)=80%	Barbariga	1	(15.3±0.4)^i^	(30.4±0.2)^g^	(51.6±0.3)^j^	(11.4±0.4)^h^	tr	(108.7±0.2)^i^	(45.4±0.3)^i^
		2	(20.6±0.2)^k^	(48.5±0.4)^m^	(93.4±0.3)^q^	(14.7±0.3)^j^	tr	(177.3±0.2)^o^	(30.3±0.2)^f^
		3	(10.6±0.3)^f^	(18.5±0.3)^c^	(30.5±0.3)^f^	(6.5±0.4)^c,d,e^	(1.5±0.2)^b,c^	(67.5±0.2)^e^	(8.2±0.2)^a^
	Lun	1	(13.5±0.2)^h^	(41.9±0.3)^k^	(63.4±0.3)^l^	(15.6±0.3)^j,k^	tr	(134.4±0.1)^l^	(23.4±0.3)^c^
		2	(17.7±0.3)^j^	(41.4±0.4)^k^	(59.9±0.1)^k^	(9.4±0.3)^g^	tr	(128.49±0.09)^k^	(30.6±0.3)^f^
		3	(8.5±0.3)^e^	(17.9±0.2)^c^	(26.4±0.4)^d,e^	(9.3±0.3)^g^	tr	(62.0±0.11)^d^	(10.5±0.3)^b^
	Hvar	1	(26.6±0.4)^m^	(56.09±0.04)^n^	(72.6±0.2)^o^	(11.4±0.3)^h^	tr	(166.7±0.2)^n^	(35.1±0.5)^g^
		2	(20.7±0.2)^k^	(36.4±0.4)^j^	(97.5±0.3)^r^	(23.6±0.4)^l^	(5.6±0.3)^e^	(183.7±0.2)^p^	(28.4±0.2)^d^
		3	(20.6±0.3)^k^	(41.6±0.3)^k^	(35.4±0.3)^g^	(5.6±0.3)^b,c^	tr	(103.1±0.2)^h^	(39.4±0.2)^h^
	Vela Luka	1	(17.5±0.3)^j^	(46.5±0.4)^l^	(62.5±0.3)^l^	(24.2±0.3)^l^	(5.3±0.2)^e^	(155.0±0.1)^m^	(27.5±0.2)^j^
		2	(20.2±0.3)^k^	(57.4±0.5)^o^	(75.5±0.3)^p^	(23.6±0.4)^l^	tr	(177.7±0.1)^o^	(29.5±0.4)^e^
		3	(10.6±0.4)^f^	(22.4±0.3)^e^	(20.4±0.4)^b^	(4.4±0.3)^a,b^	tr	(57.9±0.1)^c^	(35.5±0.4)^g^
*φ*(ethanol)=80%	Barbariga	1	(8.2±0.1)^d,e^	(28.2±0.3)^e^	(25.2±0.2)^d^	(5.5±0.2)^b,c^	(0.81±0.03)^a^	(67.9±0.2)^e^	tr
		2	(10.6±0.4)^f^	(30.3±0.1)^g^	(69.6±0.4)^n^	(7.6±0.3)^d,e,f^	(1.2±0.1)^a,b^	(119.3±0.2)^j^	tr
		3	(7.4±0.3)^c,d^	(19.8±0.2)^d^	(27.6±0.2)^e^	(5.4±0.3)^b,c^	(1.6±0.3)^b,c^	(61.8±0.3)^d^	tr
	Lun	1	(10.5±0.3)^f^	(28.7±0.2)^h^	(49.4±0.3)^i^	(6.3±0.2)^c,d^	(1.5±0.2)^b,c^	(96.4±0.1)^g^	tr
		2	(6.6±0.4)^c^	(25.6±0.4)^f^	(48.5±0.3)^i^	(8.4±0.3)^f,g^	(1.5±0.2)^b,c^	(90.5±0.2)^f^	tr
		3	(2.6±0.27)^a^	(8.12±0.09)^a^	(17.4±0.3)^a^	(4.6±0.2)^a,v^	(1.4±0.3)^b,c^	(34.1±0.2)^a^	tr
	Hvar	1	(10.8±0.2)^f,g^	(31.4±0.2)^h^	(66.6±0.3)^m^	(16.3±0.2)^j,k^	(4.6±0.2)^d^	(129.7±0.2)^k^	tr
		2	(23.3±0.1)^l^	(48.0±0.1)^m^	(74.5±0.1)^p^	(8.1±0.1)^e,f,g^	(1.5±0.2)^b,c^	(155.4±0.2)^m^	tr
		3	(10.5±0.3)^f^	(25.3±0.2)^f^	(27.7±0.2)^e^	(3.6±0.2)^a^	(1.4±0.3)^b^	(68.5±0.2)^e^	tr
	Vela Luka	1	(10.7±0.3)^f^	(35.3±0.2)^i^	(43.5±0.3)^h^	(12.2±0.2)^h^	(1.98±0.09)^c^	(103.6±0.3)^h^	tr
		2	(11.6±0.2)^g^	(36.6±0.4)^j^	(49.6±0.3)^i^	(16.6±0.3)^k^	(4.6±0.2)^d^	(118.9±0.2)^j^	tr
		3	(4.2±0.2)^b^	(14.3±0.2)^b^	(22.5±0.4)^c^	(3.5±0.1)^a^	(1.5±0.2)^b,c^	(46.1±0.2)^b^	tr

The results in each row marked with the same letter do not differ statistically at p≤0.05.

My-G=myricetin-3-*O*-glucuronide, My-Glu=myricetin-3-*O*-glucoside, My-R=myricetin-3-*O*-rhamnoside, Que-R=quercetin-3-*O*-rhamnoside,

F-G der 1=flavonol-glycoside der 1, TFG= total flavonol glycosides, tr= traces

1=early flowering stage (May, 2019), 2=early fruiting stage (August, 2019), 3=late fruiting stage (October, 2019)

In methanolic and ethanolic extracts, 5-GQA was determined as a predominant phenolic acid during the early flowering phenological stage (May). Its values in methanolic and ethanolic extracts ranged from 53.7 to 186.5 mg/L and from 22.5 to 104.4 mg/L, respectively. In both types of solvents, 5-GQA was followed by MG-Glu, 3,5-diGQA, 3,4,5-tGQA, gallic acid and caffeic acid, respectively (Table 2).

Higher mass concentrations of TPA and TFG were determined in methanolic extracts, whereas in ethanolic extracts these groups of phenolic compounds were 1.5- and 1.4-fold lower, respectively. The TPA mass concentration in methanolic extract samples ranged from 279.3 to 457.2 mg/L, and from 107.0 to 371.4 mg/L in ethanolic extracts (Table 2). According to literature data, the proper solvent choice has a significant impact on the concentration of phenolic compounds and the binary systems such as aqueous solutions of organic solvents increase the extraction yield (*28*-*30*). The highest mass concentrations of TPA in both extracts were obtained when using the samples collected during the same phenological stage (early flowering stage during May) and from the same growing location (Hvar) (Table 4).

**Table 4:** The mass fraction of phenolic compounds in methanolic and ethanolic extracts of mastic leaves influenced by the combined effects of the growing location and the phenological stage

Type of extract		*N*	*γ*(phenolic compound)/(mg/L)			
*φ*(methanol)=80%		*φ*(ethanol)=80%	
TPA	TFG	TPA	TFG
Growing location	Phenological stage		p≤0.01*	p≤0.01*	p≤0.01*	p≤0.01*
Barbariga	1	3	(279.3±0.5)^i^	(108.7±0.5)^e^	(147.6±0.7)^e^	(67.9±0.3)^d^
Barbariga	2	3	(205.2±0.5)^e^	(177.3±0.5)^j^	(73.2±0.7)^b^	(119.3±0.3)^i^
Barbariga	3	3	(136.8±0.5)^a^	(67.5±0.5)^c^	(17.3±0.7)^a^	(61.8±0.3)^c^
Lun	1	3	(183.4±0.5)^d^	(134.4±0.5)^g^	(106.9±0.7)^c^	(96.4±0.3)^f,g^
Lun	2	3	(220.0±0.5)^f^	(128.5±0.5)^f^	(127.7±0.7)^d^	(90.5±0.3)^e^
Lun	3	3	(241.9±0.5)^g^	(62.0±0.5)^b^	(169.3±0.7)^h^	(34.1±0.3)^a^
Hvar	1	3	(457.2±0.5)^l^	(166.7±0.5)^i^	(371.4±0.7)^l^	(129.7±0.3)^j^
Hvar	2	3	(352.8±0.5)^j^	(183.7±0.5)^k^	(324.1±0.7)^k^	(155.4±0.3)^k^
Hvar	3	3	(267.2±0.5)^h^	(103.1±0.5)^d^	(180.6±0.7)^i^	(68.5±0.3)^d^
Vela Luka	1	3	(416.9±0.5)^k^	(155.0±0.5)^h^	(309.6±0.7)^j^	(103.6±0.3)^h^
Vela Luka	2	3	(177.1±0.5)^c^	(177.7±0.)^j^	(157.6±0.7)^g^	(118.9±0.3)^i^
Vela Luka	3	3	(172.9±0.5)^b^	(57.9±0.51)^a^	(151.2±0.7)^f^	(46.1±0.3)^b^

Results are expressed as mean value±standard error. Values with the same letter within the column are not significantly different at p<0.01 according to Tukey’s HSD test. *Statistically significant factor at 99% confidence level. 1=early flowering stage (May, 2019), 2=early fruiting stage (August, 2019), 3=late fruiting stage (October, 2019)

Among the different phenological stages (early flowering in May, early fruiting in August and late fruiting in October), considerable variations in phenolic content were observed, indicating that the phenolic biosynthesis is significantly correlated with the plant phenological stages. The phenological stages of *P. lentiscus* in Croatia are flowering from March to late April, blooming from May to July, fruiting in July and August and fruit maturation in October. For example, flowering stage of *P. lentiscus* growing in Tunisia is during April (*31*) because phenological stages depend on the different climatic and environmental conditions. Variations in phenolic compounds depend on specific environmental conditions such as altitude, temperature and precipitation (*32*), which are characteristic for each growing location.

The obtained results show significant differences in the concentration of TPA between each phenological stage at almost all growing locations (Barbariga, Hvar and Vela Luka), and the same trend was found in methanolic and ethanolic extracts (Table 2). Generally, the highest TPA concentrations in both extracts were determined in mastic tree leaf samples harvested during early flowering in May.

Furthermore, during the vegetation from early flowering phenological stage to early fruiting phenological stage, the TPA concentrations of mastic tree leaf samples harvested at Barbariga, Hvar and Vela Luka decreased, regardless of the type of extraction solvent used. Higher TPA concentrations during the early flowering stage are probably due to the role of phenolic acids acting as precursor molecules in the biosynthesis of chalcones, flavonoids, lignans and anthocyanins (*33*). The exception was found in leaf samples from Lun, where the highest TPA concentration was found in the samples harvested during the late fruiting stage (Table 2). The reason was probably due to plant growth under local climatic conditions.

Regardless of the similar bioclimatic characteristics of the growing locations along the Adriatic coast (Table 1), *P. lentiscus* trees from Barbariga, Hvar and Vela Luka were in the form of shrubs, growing with free access to the sunlight, while trees from Lun were without direct exposure to sunlight and shaded by other trees. Linatoc *et al*. (*34*) reported that *M. indica* leaves exposed to the sun accumulated higher amounts of phenolic compounds than the shaded ones.

A consistent decrease in the TPA concentration of mastic tree leaves was observed during the early fruiting phenological stage and the lowest during the late fruiting phenological stage. Generally, the mass concentrations of TPA in both methanolic and ethanolic extracts were lower at later phenological stages except in the samples from Lun. The samples from Hvar had the highest mass concentrations of TPA in both extracts during the early flowering stage (457.2 and 371.4 mg/L) and the lowest in the samples from Barbariga growing location (136.8 and 17.3 mg/L) during the late fruiting phenological stage (Table 2).

According to the data in Table 1, Hvar was characterised by the highest altitude (330 m) and average temperatures, high average insolation and low precipitation through all the phenological stages. Barbariga growing location had the lowest measured altitude (4 m).

Altitude is an important parameter that affects phenolic content and it is characterised by air temperatures and solar exposure. As the altitude increases, the plant becomes more exposed to ultraviolet radiation. As a result, plant defence mechanism activates, triggering the production of secondary metabolites such as phenolic acids and flavonoids, which have the capacity to absorb UV radiation (*35*). It is highly possible that the combined influence of these factors triggered a response in the plant, resulting in an increased biosynthesis of phenolic compounds. Our results suggest that mass concentrations of phenolic compounds considerably differ depending on the differences in altitude, which is in accordance with other research (*35*, *36*).

The most significant decrease in TPA among phenological stages was observable in the samples from Vela Luka. The decrease in methanolic extracts was 57.51%, and in ethanolic extracts 49.08%. Unlike the samples from Vela Luka, in samples from Lun, the TPA increased from the early flowering to the early fruiting phenological stage, by 20.00% (Table 2).

According to the statistical analysis, individual influence of extraction solvent, growing location and phenological stage as well as the influence of the interaction of growing location and phenological stage had significant impact on the TPA concentration of mastic tree leaf samples (p<0.01) (Table 4 and Table 5). The highest TPA concentration was determined in the samples harvested in Hvar during the flowering phenological stage, extracted by 80% methanol.

**Table 5:** The mass concentration of phenolic compounds in mastic tree leaf extracts inﬂuenced by extraction solvent, growing location and phenological stage

Extraction parameter	*N*	*γ*(biologically active compound)/(mg/L)	
TPA	TFG
Solvent		p<0.01*	p<0.01*
*φ*(methanol)=80%	36	(259.2±0.2)^b^	(126.9±0.1)^b^
*φ*(ethanol)=80%	36	(178.0±0.2)^a^	(91.0±0.1)^a^
Growing location		p<0.01*	p<0.01*
Barbariga	18	(143.2±0.2)^a^	(100.4±0.2)^b^
Lun	18	(174.9±0.2)^b^	(90.9±0.2)^a^
Hvar	18	(325.6±0.2)^d^	(134.5±0.2)^d^
Vela Luka	18	(230.9±0.2)^c^	(109.9±0.2)^c^
Phenological stage		p<0.01*	p<0.01*
1	24	(284.0±0.2)^c^	(120.3±0.2)^b^
2	24	(204.7±0.2)^b^	(143.9±0.2)^c^
3	24	(167.2±0.2)^a^	(62.6±0.2)^a^

Results are expressed as mean value±standard error. Values with the same letter within the column are not significantly different at p<0.01 according to Tukey’s HSD test. *Statistically significant factor at 99% confidence level. 1=early flowering stage (May, 2019), 2=early fruiting stage (August, 2019), 3=late fruiting stage (October, 2019)

The TFG concentration in methanolic extracts ranged from 57.9 to 183.7 mg/L and in ethanolic extracts from 34.1 to 155.4 mg/L (Table 3). The most abundant flavonol glycoside in methanolic and ethanolic extracts was My-R, followed by My-Glu, My-G, Que-R and derivative of flavonol glycoside, respectively. Catechin was determined (8.1-45.4 mg/L) only in methanol leaf extracts, while in the ethanol extracts it was not identified (Table 3).

Contrary to TPA, almost all mastic tree leaf extracts had the highest TFG concentration in the early fruiting phenological stage, with the exception of leaf extracts from Lun growing location (early flowering phenological stage) (Table 3). It is well known that flavonoids and phenolic acids have important roles in plant defence mechanisms (*37*), and previously we explained unfavourable tree position in Lun. According to Jakovljevic *et al*. (*38*) low flavonoid concentrations of *Chelidonium majus* were determined at the beginning of the flowering phenological stage, which increased during early fruiting phenological stage.

Considerable differences in TFG concentrations were also observed between early flowering and fruiting stages among all growing locations. The concentrations of TFG in methanolic extracts from Barbariga and Lun decreased from early flowering to early fruiting stage by 63.15 and 4.36%, while in ethanolic extracts the decrease was 75.64 and 6.03%, respectively. At Hvar and Vela Luka TFG increased from early flowering to early fruiting phenological stage. In methanolic extracts, the increase was 10.20 and 14.66%, and in ethanolic extracts 19.83 and 14.81%, respectively. The obtained results can be attributed to the stressful conditions caused by higher average precipitations measured in locations Barbariga (84.20 mm) and Lun (48.70 mm), unlike other locations (Hvar and Vela Luka), which had lower average precipitation (19.30 to 33.00 mm) during May (Table 1). Gull *et al*. (*39*) reported an increase in the biosynthesis of TFG in *C. spinosa* and *C. decidua* during the month with the highest precipitation (September).

The lowest TFG concentration (34.1-103.1 mg/L) of mastic leaf extracts was determined during the late fruiting phenological stage (Table 3). The phenological stage, growing location and type of extraction solvent significantly affected TFG concentration in mastic tree leaf samples, so did the combined influence of interaction of growing location and the phenological stage (p<0.01) (Table 4 and Table 5). Only in methanol extracts obtained from the samples harvested at Barbariga and Vela Luka during the early fruiting stage, there was no significant combined effect of growing location and phenological stage on TFG concentrations. Also, there was no significant combined influence of growing location and phenological stage on the TFG concentration in ethanol extracts obtained from the samples harvested at Barbariga location during flowering stage (May) and Hvar during the late fruiting stage (October), as well as on TFG of Vela Luka and Barbariga samples during the early fruiting stage (Table 5).

The highest concentrations of TFG and TPA in mastic tree leaves were determined in the samples extracted with 80% methanol, both harvested at Hvar but during different phenological stages.

Our results are in accordance with the study of Liu *et al*. (*36*) who reported a positive correlation between the increase of altitude, sunshine duration and total flavonoids of *Lycium chinense* stems and leaves due to the increase in ultraviolet radiation. Kobayashi *et al*. (*40*) reported a negative correlation between the average air temperature and phenolic content of *Ipomoea batatas*, suggesting that lower temperatures increased the total phenolic content.

Higher variations were noticed in catechin concentrations in methanol leaf extracts where leaf samples from Barbariga had the highest catechin concentration ((45.4±0.2) mg/L) during the early flowering phenological stage, leaves from Lun in early fruiting phenological stage ((30.6±0.3) mg/L) and leaves from Hvar ((39.4±0.2) mg/L) and Vela Luka ((35.5±0.4) mg/L) during the late fruiting stage.

According to the study of Aoussar *et al*. (*41*), the highest phenolic content of *Pseudevernia furfuracea*, *Evernia prunastri* and *Ramalina farinacea* grown in Morocco was determined during late winter and spring when temperatures were lower and precipitation rates higher. Numerous studies have also confirmed that the phenolic content can be strongly influenced by climatic conditions, harvesting location, time and phenological plant stage (*42*, *43*).

## CONCLUSIONS

The results obtained in this study show that mastic tree leaves harvested at four growing locations along the Adriatic coast (Barbariga, Lun, Hvar and Vela Luka) during three phenological stages (early flowering, early fruiting and late fruiting) might be considered as a significant natural source of diverse phenolic compounds (phenolic acids and flavonoids). Total of seven phenolic acids and five flavonol glycosides were quantified, with 5-*O*-galloyl-quinic acid being the predominant compound among phenolic acids, and myrcetin-3-*O*-rhamnoside among flavonol glycosides. The phenolic mass concentration in mastic tree leaves was significantly affected by the environmental factors, growing location and phenological stages.

Among the samples harvested at four different growing locations, samples harvested at Hvar growing location had the highest concentration of phenolic compounds during all phenological stages due to the environmental conditions of the growing location such as altitude, average precipitation and temperature, which showed a positive correlation to total phenolic acids (TPA) and total flavonol glycosides (TFG). Our findings highlight the importance of growing conditions and plant phenological stage to select mastic tree leaves with the maximum yield of phenolic compounds. The early flowering phenological stage in May was the most appropriate period for the harvesting of *P. lentiscus* with the maximum yield of TPA, while harvesting at early fruiting stage in August was the most appropriate period for maximum yield of TFG. From the obtained results, it can be seen that besides the growing location and phenological plant stage, the choice of extraction solvent also plays an important role in determining the quality of mastic tree leaf extracts. Regarding the extraction of phenolic compounds, 80% methanol was a more effective solvent than 80% ethanol. These findings may be useful in highlighting mastic tree leaf extracts as a promising source of natural antioxidants in food and pharmaceutical industries, therefore, further research is necessary.
